# State of the art in EEG signal features of mindfulness-based treatments for chronic pain

**DOI:** 10.1007/s10072-025-08145-3

**Published:** 2025-03-29

**Authors:** D. Duran, P. Arpaia, G. D’Errico, L. Grazzi, P. Lanteri, N. Moccaldi, A. Raggi, R. Robbio, E. Visani

**Affiliations:** 1 Neurological Institute Carlo Besta, Milan, Italy; 2 Department of Electrical Engineering and Information Technology, University of Naples Federico II, Naples, Italy; 3 Department of Applied Science and Technology, Polytechnical University of Turin, Turin, Italy

**Keywords:** Electroencephalography, Chronic pain, Mindfulness, Non-pharmacological treatment

## Abstract

A systematic review of electroencephalographic (EEG) correlates of Mindfulness- based treatment for chronic pain is presented. Recent technological advances have made EEG acquisition more accessible and also reliable. EEG monitoring before, during, and after treatment might support efficacy assessment and enable real- time adaptive intervention. The preliminary research extracted 131 papers from 6 scientific search engines. The application of the exclusion criteria led to the selection of 4 papers, indicating that the topic is still unexplored and further investigations are required. The collected papers exhibited great variability making challenging the comparison, nevertheless promising EEG correlates emerged. In particular, pain-related evoked potentials correlate with Mindfulness-Based treatment. EEG source analysis revealed the prevalent involvement of regions modulating emotional responses. In addition, higher baseline theta power was associated with greater improvement in depression when Mindfulness-based treatments are administered. This last result makes EEG also suitable for evaluating which patients can benefit most from mindfulness-based treatments.

## Introduction

According to the revised International Association for the Study of Pain (IASP) definition [1], pain is ”an unpleasant sensory and emotional experience associated with, or resembling that associated with, actual or potential tissue damage”. When it persists or recurs for more than 3 months, it is classified as chronic. Around 20% of the world’s population suffers from chronic pain (CP), which is associated with psychological, biological or social factors. The IASP classification of CP [2], included in the 11th edition of International Statistical Classification of Diseases (ICD-11) [3], distinguishes between primary and secondary CP. Primary CP is the direct symptom of a condition, e.g. fibromyalgia, chronic migraine or nonspecific low-back pain; secondary CP is characterized by symptoms that originate from another primary disease, e.g. chronic cancer pain, or sequelae of traumas [4].

CP management requires a multimodal approach, which incorporates pharmacological and non-pharmacological treatments [5, 6]. The latter include psychological interventions to support patients in coping with CP-related difficulties, by enabling managing the factors that hinder daily functions, exacerbate discomfort, and amplify painful experiences [7]. Mindfulness-based interventions target the cognitive, affective, and sensory dimensions of pain by cultivating an awareness of the present moment without judgment [8], and are currently used for the treatment of pain in several contexts [9]. The Mindfulness-Based Stress Reduction (MBSR) is an 8-week structured group program (one session per week of 2.5 hours). The goal is to teach patients a new approach to managing their illness, emphasizing self-regulation of pain and negative emotions to enhance their sense of control. The program is used for various CP conditions and positively impacts stress, depression and anxiety [10]. Mindfulness-Based Cognitive Therapy (MBCT), consists of 8 weekly sessions, 2 hours each. It combines principles from Cognitive Therapy and Mindfulness Meditation, with the aim to increase awareness of cognitions, fostering a mindful and accepting approach [11]. Mindfulness Based Pain Management (MBPM) [12] consists of a total of 20 hours of training, spread over 8 weeks, with each session lasting 2.5 hours. The MBPM program instructs participants to engage in Mindfulness training to disrupt the reactive pain cycle that exacerbates physical and emotional stress. Participants cultivate the practice of ’breathing into’ difficult experiences to reduce resistance to pain and release tension.

Recent technology advancements (Extended reality, wearable sensors, etc.) pro- vide insights to enrich and potentiate Mindfulness-based therapeutic practice. The user can immerse himself in a simulated environment that can be customized. Virtual Reality (VR) can be exploited to implement an adaptive experience with the use of biofeedback, leading to greater concentration toward the present moment. In addition, the VR environment allows the subjects to immerse themselves in any scenario while remaining in the clinical or home setting. VR has been found to reduce levels of anxiety, stress, depression and CP by increasing user engagement [13]. Therefore, it could lead to successful Mindfulness practice. The exploitation of EEG signals monitoring during Mindfulness treatment opens new opportunities for real-time therapy personalization. Neurocorrelates of Mindfulness were widely studied in the literature [14, 15]. A review targeting Mindfulness, pain and their neurocorrelates [8] found that somatosensory regions were more activated in expert meditators than in novices, whereas a lower activity of cognitive-affective regions (e.g., prefrontal region) was present, which in novices was less evident. This suggests a potential dissociation between the cognitive-affective and sensory dimensions of pain in experienced meditators. Moreover, an increased modulation of alpha power occurred in expert meditators in order to suppress the sensory input processing. The increased activity in the secondary somatosensory cortex (S2) and posterior insula during meditation compared with rest in experts could reflect increased attention toward bodily sensations and decreased unpleasantness of pain. In contrast, decreased activity in the medial prefrontal cortex (mPFC) and anterior cingulate cortex (ACC) in expert meditators could reflect greater emotional control of painful stimuli. A lower pain ratings in both classes of meditators, as compared to the control class, was also reported in several studies cited. Structural and functional changes, such as increased S2 thickness and increased parietal alpha power during painful stimuli, were prevalent in experienced meditators, which were not found in novice meditators. Wang’s review investigated the short and long-term effects of Mindfulness meditation on pain-related brain processes by comparing the neural activities in beginners and experts meditators [8], and also it focused on multimodal approaches, including both EEG/EMG monitoring and the use of brain imaging through magnetic resonance (MRI/fMRI).

Specifically, experienced meditators showed an increased thickness of the somatosensory cortex and dorsal anterior cingulate cortex (dACC), linked to superior pain tolerance. In addition, they showed less activation in prefrontal regions and more activation in somatosensory regions during meditation. What clearly emerged from these studies is that Mindfulness practices provides greater control over pain processing, promoting relaxation states and less tension, as evidenced by an increase in alpha wave power in the parietal and occipital regions, as well as brain restructuring (e.g. increased thickness), which also allowed for a reduction in the impact of CP on emotions. Indeed, the difference in activation or deactivation of brain areas between experienced and novice meditators indicates effective neuroplasticity promoted by Mindfulness practice. In fact, somatosensory or cognitive-affective areas are also associated with pain processing and modulation of painful sensations [16]. The study in [16] refers to a “pain matrix”, identified as a brain network related to pain processing, and it includes: somatosensory cortices and thalamus associated with the localization of pain, and discrimination of its intensity whereas, the insula and anterior cingulate cortex are related to the emotional aspects and attention toward pain. The joint activation of these regions was found to underlie conscious perception, attentional modulation and control [17]. Furthermore, the pain matrix was considered to reflect a system that brings attention toward the occurrence of a specific sensory event which leads to reaction to a possible danger, not only, therefore, capable of transforming painful sensory input into pain conscious perception [18]. This could be the foundation of a reassessment of the painful stimulus and a remodulation of the focus on the event associated with it through the practice of mindfulness. Nevertheless, the specific and unique association between painful stimuli and the brain areas defining the pain matrix is still debated in the literature, as these areas are also associated with other cognitive processing, and the function of different regions depends on the context in which the stimuli are provided [17, 19–21]. In [16], several studies are reported highlighting the neuroplasticity of precisely these areas of the brain by long-term meditation. In particular, an increase in cortical thickness was found mainly in the primary and secondary somatosensory cortex, prefrontal cortex, posterior parietal cortex, temporal gyrus, anterior cingulate cortex, and hippocampus, with increased connectivity between the posterior cingulate cortex, prefrontal cortex, and hippocampus, suggesting an increase in neuronal volume in these regions, which probably contributes to improved cognitive and emotional processing. An increased gray matter volume was found in areas related to emotion regulation and pain processing, such as the hippocampus, amygdala, and thalamus, but even, for instance, in the orbitofrontal cortex and posterior parietal cortex. Also, an improved anatomical connectivity of the white matter was observed, suggesting increased efficiency in neural connections. In addition, experienced meditators showed less pain anticipatory activity in the amygdala and exhibited faster neural habitation in response to pain in the mid-cingulate cortex by analyzing the blood-oxygen-level-dependent (BOLD) signal, probably resulting from a reduction in the emotional impact of anticipation of pain.

Thus, variations in the CP neurophysiology are related to changes in brain’s functional and structural characteristics. CP also leads to changes in the neural networks involved in pain modulation. The dual nature of CP (emotional and sensory) results in a relationship between pain and the mechanisms of somatosensory areas on one side, and between pain and the affective and cognitive mechanisms of other areas on the other side. For instance, nocebo hyperalgesia is an effect of increased intensity of perceived pain caused by anticipatory pain anxiety, promoting a release of cholecystokinin and pain transmission [22]. For this reason, the dissociation of CP related to sensory pain from that due to anxious or depressive states remains quite complex to achieve. An examination of the neurophysiological changes in CP, employing Mindfulness, would be useful to test the plasticity of “actual or potential tissue damage” and/or brain-level reprocessing of a response to the painful insult. Although clinical evidence exists for the effectiveness of Mindfulness in reducing pain perception or frequency (e.g [23]) EEG-based analyses regarding the efficacy of Mindfulness treatments in CP are currently lacking.

Hence the need for a review that focuses on a specific and detailed analysis of changes in EEG features related to pain in response to Mindfulness interventions in.

individuals with CP. Therefore, the aim of this review is to investigate EEG features related to objective effects on CP produced by Mindfulness-based treatments. In this regard, the results of studies on EEG related to Mindfulness-based treatment for CP are described and assessed.

## Methods

### Search strategy

**Fig. 1:**
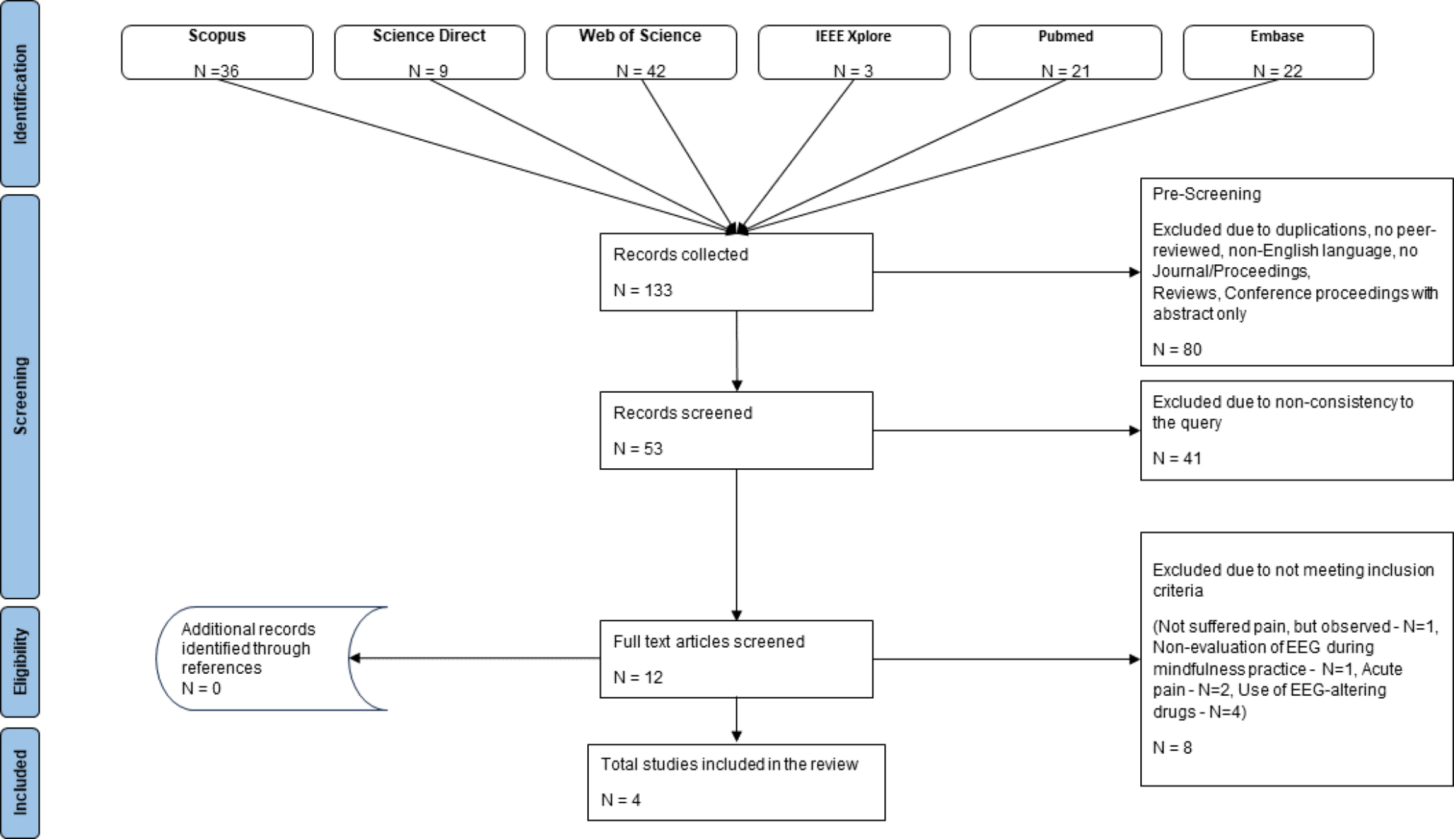
PRISMA flow diagram of selected studies

The databases queried for the literature search were PubMed, Scopus, Embase, ScienceDirect, Web of Science, and IEEE Xplore. The search on each database was conducted with the following keywords in combination and with the restriction to article title, abstract and keywords: Mindfulness AND (eeg OR electroencephalography OR electroencephalographic) AND (migraine OR pain OR ache OR headache OR backache OR hyperalgesia OR causalgia OR hyperpathia OR allodynia OR neuralgia OR radiculopathy OR fibromyalgia). No date limitation was imposed. In order to identify further material, a manual search of the selected study references was carried out. The paper extraction process was conducted according to PRISMA recommendations [24] in a systematic and transparent way. The PRISMA flow diagram of the selected studies is reported in Fig. 1. In the pre-screening stage, all duplicates,

non-peer-reviewed articles, non-journal or conference proceedings, reviews, articles in proceedings of which only conference abstracts exist, and articles that were not written in English language were excluded. Subsequently, items that were not consistent with the purpose of the research were excluded. Finally, the eligibility stage was applied, excluding (i) articles that referred to observed pain (i.e. empathy for the pain that other people suffer), (ii) articles in which the EEG signal was not assessed in relation to Mindfulness, and lastly (iii) articles with subjects who did not suffer from CP, but they were induced acute pain, articles in which the subjects took drugs that could alter the EEG signal. The whole procedure was characterized by multi- author checking on the abstract and full-text of the papers found, to assess further congruence with the research question. Articles abstract check was conducted by a subgroup of authors, while articles full text check was conducted by all review authors. In case of disagreement, all the authors were involved to discuss further and attempt convergence.

### Data analysis

All articles that were not excluded from the eligibility phase were submitted for a descriptive analysis with regard to the findings obtained from each of them. Specifically, potential trends in EEG features resulting from the treatment were assessed, determining whether a significant difference in these features between pre- and post- treatment was observed. Additionally, general clinical outcomes were examined to summarize whether these studies reported specific evidence of mindfulness-based treatments affecting both neurophysiological and clinical outcomes.

## Results

One hundred and thirty-three articles resulted after database search, twelve were subjected to full-text screening, and four were submitted for systematic review [10, 11, 25, 26]. Table 1 reports the included studies, defining for each one the type of article, the type of Mindfulness treatment employed, the number of sessions and duration of each session, the number of subjects and the type of participants, the grouping of participants, their age, and the pathology they suffer from. Each article employed a different Mindfulness protocol. Day et al. (2020) [11] and Schmidt et al. (2015) [10] conducted the analysis on only participants with chronic low back pain (CLBP), while Brown et al. (2013) [26] considered different types of conditions, including CLBP. In general, grouping of participants into a Mindfulness intervention group and a control or other treatment group was applied in all studies, except in the study of Schmidt et al. (2015).

Table 2 shows, for each article, the EEG signal acquisition device, the number of channels, when the signal was acquired with respect to the treatment, and at last the scales used and the questionnaires administered. As can be observed, the number of channels used to acquire the signal was in most studies about 60, with different devices. Moreover, in all cases the EEG signal was acquired at both pre-treatment and post- treatment. Most of the studies implemented pain assessment by exploiting different scales, e.g. the Numerical Rating Scale (NRS), Visual Analogue Scale (VAS), or Pain Perception Scale (PPS). Moreover, psychological assessment was such as that inherent to depression. Two studies (Day et al. (2020) [11], Brown et al. (2013) [26]) also employed questionnaires related to measuring present-moment attention and Mindfulness, like.

**Table 1:** Recruitment and treatment protocol

Author (year)	Mindfulness Treatment (sessions)	Sample (typology)	Grouping	Age (years)	Age (years)	
Ramalingam et al. (2018) [25]	VAMDB (6-week session)	12 (athletes)	Intervention (N=4); Non-intervention (physiotherapy) (N=4); Control (N=4)	18–25	Chronic Pain	Ankle
Day et al. (2020) [11]	MBCT (8-week session); MM (8-week session)	69	MBCT; MM; CT	≥ 18	Chronic Back Pain	Low
Schmidt et al. (2015) [10]	MBSR (8-week session)	21	No grouping	37–57	Chronic back pain	low
Brown et al. (2013) [26]	MBPM (two 8-week ses- sions)	40	MBPM (N = 15); Control (N = 15)	38–58 (MBPM); 33–57 (Control)	Musculoskeletal pain	

VAMDB Video Assisted Mindfulness Deep Breathing, MBCT Mindfulness-Based Cognitive Therapy, MM Mindfulness Meditation, CT Cognitive Therapy, MBSR Mindfulness-Based Stress Reduction, MBPM Mindfulness-Based Pain Management

Five Facet Mindfulness Questionnaire-Short Form (FFMQ-SF) and Mindful Attention and Awareness Scale (MAAS). The EEG features extracted and analysed, as well as the region of the scalp from which they were extracted, information about the statistical analysis, namely the goal, tests and observed parameters, are presented in Table 3.

**Table 2:** Material and methods

Author (year)	Hardware	Channel number	Time of acquisition	Scales and questionnaires
Ramalingam et al. (2018) [25]	Emotiv EPOC	14	Pre- and post-treatment	Pain score
Day et al. (2020) [11]	Waveguard	64	Pre- and post-treatment	13-item PCS; FFMQ-SF; PROMIS; NRS;
Schmidt et al. (2015) [10]	ActiCap	60	Enrolment, pre- and post-treatment	EQ-5D; SF-12; BSI; HADS; PPS; VAS; FLZ;
Brown et al. (2013) [26]	Neuroscan	61	Pre- and post-treatment	Short-Form McGill Pain Questionnaire; Short-Form 36 health survey; Pain Stages of Change questionnaire; Sur- vey of Pain Attitudes; MAAS

PCS Pain Catastrophizing Scale, FFMQ-SF Five Facet Mindfulness Questionnaire-Short Form, PROMIS Patient-Reported Outcomes Measurement Information System, NRS Numerical rating scales, EQ-5D EuroQol Quality of Life Questionnaire, SF-12 Twelve-Item Short Form Health Survey, BSI Brief Symptom Inventory, HADS Hospital Anxiety and Depression Scale (HADS), PPS Pain Perception Scale, *VAS* Visual Analogue Scales, FLZ Questions on Life Satisfaction, MAAS Mindful Attention and Awareness Scale

.

In the study presented by Ramalingam et al. (2018) [25], a customized Mindfulness- based intervention is proposed, namely the Video Assisted Mindful Deep Breathing (VAMDB). VAMDB consisted of watching 3-minute videos to instruct participants on deep breathing, for 6 weeks. The same deep breathing exercise was performed.

**Table 3:** EEG features analyzed and statistical analysis details

Author (year)	Features extracted	Region of the scalp	Goal of analysis	Type of analysis	Observed parameters
Ramalingam et al. (2018) [25]	Delta, theta, alpha, beta, gamma abso- lute powers	Frontal (AF3, AF4), Occip- ital (O1, O2), Right Tem- poral(F4, F8, FC6, P8, T8), Left Temporal (F3, F7, FC5, T7, P7)	Assess differences between groups and differences between pre- and post- intervention	Analysis of covariance (ANCOVA)	p-value
Day et al. (2020) [11]	Theta and Alpha relative power	Centro parietal (CPz), Antero Frontal (AFz)	Evaluation of the interaction between the moderator and MM vs. MBCT and between the moderator and CT vs. MBCT.	Omnibus test series of regions of significance (OGRS), Johnson- Neyman significance limit for identifying regions	p-value, effect size
Schmidt et al. (2015) [10]	Peakfrequency, Peak power, ROI^∗^ Center of Gravity (average value), ROI Overall power	Not reported	Evaluation of the correlation between pre and post inter- vention EEG feature varia- tion and clinical outcome	Correlationanalysis with Spearman’s rank correlation coefficient and Pearson’s correla- tion coefficient	p-value, effect size
Brown et al. (2013) [26]	Early and late antic- ipation periods^∗∗^, P2 peak	Parietal and occipital (POz, Pz, Oz, Po3, PO4), Central and Parietal (CPz, Cz, Pz, CP1, CP2), Central and fronto central (Cz, FCz, CPz, C1, C2)	Evaluation of differences between the groups and in the two sessions	Nonparametric repeated-measures ANOVA	p-value, effect size

∗ A frequency Region of Interest (ROI) of *±*2 the standard deviation of the mean peak frequency was adopted

∗∗ Two 500 ms time periods of the anticipatory brain response were defined: *early anticipation period* ranging from − 2500 to -2000 ms before the laser stimulus, and *late anticipation period* ranging from − 500 to 0 ms before the laser stimulus

autonomously by the participants for at least 6 days per week. Regarding statistical analysis conducted on the post-intervention data revealed significant differences between VAMDB and the physiotherapy interventions in the occipital area for absolute power in delta band, theta band, alpha band, gamma band and beta band. The modulation of absolute power in the occipital area could be derived from visual processing and could be related to attentional changes during pain relief. Absolute power in beta band also defined significant differences between VAMDB and physio- therapy in the right temporal region, which could be associated with cognitive processing and alertness. In fact, modulation of absolute beta-band power could be related to sustained attention. In all the considered bands for the respective significant regions, the mean absolute power for the physiotherapy intervention was higher than that obtained in VAMDB or the control group, and that of the control group was still higher than the mean power value for the VAMDB intervention. Regarding pain assessment, there was a significant difference in this case specifically between VAMDB and the control group. The authors of the study considered that the beta-wave response in the right temporal and occipital region could be based on the participants’ brain alertness due to the reduction of pain during the post-intervention measurement. In the analysis of the difference between pre- and post-intervention, a similar trend occurred in all regions with a decrease in absolute powers in alpha, beta and gamma bands and increase in baseline assessment in delta and theta, probably indicating a less cognitively active and more relaxed state.

The study conducted by Day et al. (2020) [11] is based on the Limit, Activate, and Enhance (LA&E) model [27]. According to this model, different types of moderators acting in the treatment-outcome clinical relationship are discriminated: (i) the basic ”weaknesses” that need to be limited (e.g., pain catastrophizing), (ii) ”strengths” that can be enhanced (e.g., Mindfulness) to improve outcomes, and (iii) other attributes that need to be activated (e.g., the state of focus and attention which is measured with EEG). In detail, the authors considered as starting hypotheses how low relative powers in alpha and theta bands may be moderators of activation in MM and MBCT, compared with CT. The results of significance analysis in the moderation of NRS and PROMIS scales for alpha and theta relative powers, in terms of p-value, revealed no significant difference in the moderation of NRS - pain. Conversely, a significant difference was found for theta relative power in the moderation of PROMIS - depression when moderator and MBCT vs. CT interaction was observed. Specifically, higher theta relative power at baseline was found in the MBCT group which was associated with improvement in depression. On the other side, considering the high baseline pain catastrophizing as a ”Limit” moderator, a significant interaction term for the PCS effect and the MM vs. MBCT condition was found, highlighting that the difference in pain intensity improvement between these two groups depended on the PCS score value at baseline. In the MM group, individuals with a higher PCS score at baseline experienced greater improvement in pain intensity. Conversely, in the MBCT group, those with a lower PCS score at baseline showed greater improvement in pain intensity. In summary, Mindfulness treatments for CP did not result more clinical effective on individuals with low baseline values of relative power in alpha and theta band. On the contrary, evidence emerges that high relative power in theta band correlates with the outcome relative to depression in case of Mindfulness-based treatment. The most evident result is that the effectiveness of each treatment varies among individuals, indicating that certain personal characteristics and cognitive or brain predispositions could result in greater or lesser response to treatment.

Schmidt et al. (2015) [10] considered the theory of thalamocortical dysrhythmia (TCD) [28, 29], according to which an enhancement of pain should be related to: (i) shift of peak frequency and center of gravity to a higher frequency, and (ii) general reduction of overall power and peak power. By comparing the EEG features before and after the intervention, the results obtained showed that all the parameters shifted in the opposite direction from that estimated, and none of them was statistically significant. The change in mean pain perception over the past 4 weeks was also correlated with changes in TCD-related EEG features between the two instants of measurements (before and after surgery). In such case, all correlations were in the hypothesized direction, with pain improvement resulting in lower overall power, lower peak power, and increased peak frequency and center of gravity. Nevertheless, after multiple analysis corrections, no significant correlations remained. In the end, EEG analyses showed no significant pre- and post-MBSR intervention changes.

In the study by Brown et al. (2013) [26], acute pain was induced with a laser on subjects with CP in 2 sessions to increase experimental reproducibility concerning pain intensity. In particular, ERP sources related to pain anticipation and experienced painful stimuli were examined. In addition, the sources of anticipated and pain-evoked potentials were also identified using the imaging approach to source reconstruction. The MBPM group exhibited a decrease in the amplitude of pain-related evoked potentials and ERP, in contrast to the control group. A statistically significant difference was found comparing the late anticipation period between groups and sessions at both Cpz and Cp2 electrode This was indicative of decreased neural activity in the intervention group compared with the control group. Even at C2 electrode a statistical significant difference emerged for the P2 peak Furthermore, in the post-MBPM intervention, dorsolateral prefrontal cortex (DLPFC) and S2 regions showed less deactivation during pain anticipation, indicating a more stable brain activity in response to pain expectation.

## Discussion

The aim of the review was to identify EEG correlates in subjects with CP resulting from Mindfulness-based treatments. Four heterogeneous studies were selected, which used different Mindfulness-based approaches, different EEG features and had different research purposes. Despite such heterogeneity, the studies pointed out that pain-related evoked potentials correlate with Mindfulness-Based treatment, and revealed a prevalent involvement of regions modulating emotional responses.

CP may affect EEG features. For instance, in [30], hyper activation in high theta (6–9 Hz) and low beta (12–16 Hz) bands, in pain-related brain areas (e.g. pre-frontal and posterior inferior parietal cortex, or in the primary somatosensory cortices, secondary somatosensory cortices, or anterior cingulate cortex), were found in patients with CP, and pain processing arises from both sensory and affective-cognitive mechanisms, which can be modulated by Mindfulness. The role of meditation in the association between pain sensitivity and structural changes was investigated and reported in [31]. Intensive mindfulness practice resulted in a reduced activation of areas related to emotional and cognitive appraisal of pain (e.g., amygdala, hippocampus and prefrontal cortex). A reduced connectivity was also found between regions involved in executive function and cognitive processing of pain. EEG features correlating with CP and Mindfulness-based treatment might be useful for providing daily clinical routines and ensuring better treatment performance. The EEG biomarkers, related to both pain management and improved Mindfulness, are mainly connected to changes in alpha and theta wave activity, related to relaxation, emotional and cognitive control, but also to ERPs reflecting control of attention and sensory processing [15, 32]. Analysis of ERP studies reveals that CP patients exhibit alterations in early allocation of attentional resources and later engagement of motivation-based cognitive processes when processing important information for the body.

Included studies provide a promising overview of the EEG correlates and clinical outcomes of patients with CP undergoing Mindfulness-based treatments. Highly promising results concerning changes in pain-related evoked potentials and ERP correlated with Mindfulness treatments are presented in [26]. Mindfulness practice promoted a better management the emotional aspect of pain anticipation, avoiding the typical reduction of activity in brain areas that are involved in emotion regulation and cognitive control. The patients experienced improvements in mental health and control over pain, despite no significant reduction in pain intensity, which underlies the complex interaction between Mindfulness, neural activity, and emotional processes. Day et al. [11] adopted the LA&E model and explored the moderation effects of alpha and theta powers in Mindfulness treatments: they provided evidence for a significant correlation between relative power in the theta band and modulation of depression among participants.

In terms of the spatial domain, the experimental group in Brown’s study [26] showed a decrease both at Cpz and Cp2 in the amplitude ERP related to anticipation and pain, contrasting with the control group. Furthermore, in the post-MBPM intervention, the regions of the DLPFC and S2 showed less deactivation during pain anticipation, indicating a more stable brain activity in response to pain expectation. These results confirm the feasibility of identifying EEG markers of Mindfulness-based treatment effects. However, there are intrinsic issues with Brown’s study [26]. In fact, the study does not focus on CP but administers acute pain stimuli to patients with CP in order to increase experimental reproducibility of pain intensity. The identified neurocorrelates are acquired in a highly structured experimental setup and require synchronization with the source of pain to be detected. All of this impacts on the ecological validity of the study. Moreover, the ERP is a feeble signal, requiring repeated acquisition under synchronized conditions for averaging the background noise. Although Brown’s results are promising, these data are hardly applicable in daily clinical routine as ERP results are difficult to use in the single case: there is a poor replicability even in the same subject, dependent, for example, on attentional levels which are not easily controlled. Certainly, the most relevant result of this overview deals with the utility of EEG signals in Mindfulness as biomarkers of pain modulation and control as intended of control over emotional states related to pain perception rather than actual pain sensation.

Some limitations have to be acknowledged. First, although we engaged in an extensive literature search, we cannot completely exclude that some relevant papers were not identified. We were aware of the paucity of literature in the field, and therefore employed any possible measure to expand to the widest extent possible the amount of candidate papers, e.g. using a comprehensive set of key-words for pain-related conditions and manual search among the selected study references. Second, we could not handle the heterogeneity of results beside a pure description: in fact, the selected papers varied in study designs, disease category, EEG device, and also in the EEG features considered, making a comparative analysis of results challenging. The introduction of guidelines and standards will become increasingly necessary, and future research should adopt experimental paradigms inspired by previous works to reduce the variability across studies. Further research should investigate the long-term effects of Mindfulness-based treatment on EEG patterns, for enabling the quantitative assessment of the intervention effectiveness. Moreover, profiling individual EEG pattern variation could improve the design of more targeted, customized, and effective approaches for CP management.

## Conclusion

We conducted a systematic review to explore the emerging yet promising landscape of EEG correlates in individuals undergoing Mindfulness-based treatments for CP. Despite the methodological heterogeneity and variation in clinical populations across the studies reviewed, several noteworthy insights have surfaced. There is a potential interplay between cognitive-emotional dimensions and pain perception, indicating that Mindfulness interventions may exert beneficial effects by modulating emotional responses to pain. Alterations in ERP sources are associated with pain anticipation and responses to painful stimuli: these specific neurocorrelates reveal a decrease in the amplitude of potentials related to pain anticipation and actual pain, suggesting a neural basis for the observed improvements in emotional regulation and cognitive control.

EEG is a promising neurophysiological biomarker of the effect of Mindfulness meditation, possibly demonstrating brain-level influence and modulation of pain. However, while these results hold promise for the development of instrumental indicators and personalized treatment approaches, a challenge persist regarding the clinical applicability due to the required complex experimental setup. Future research should address these limitations by adopting standardized methodologies, conducting long-term follow-ups, and exploring individual variations in EEG patterns to tailor interventions.

## Declarations

### Authors' declaration

The submitted work is original and has not been published elsewhere in any form or language, either partially or in full. The manuscript is not under consideration by any other journal for simultaneous publication. Proper acknowledgements to other works have been given, and no data, text, or theories by others are presented as if they were the authors' own. Quotation marks are used for verbatim copying of material, and permissions were secured for any copyrighted material. The results and discussions presented in this review are honest, clear, and without fabrication, falsification, or inappropriate data manipulation.
